# The Globular State of the Single-Stranded RNA: Effect of the Secondary Structure Rearrangements

**DOI:** 10.1155/2015/295264

**Published:** 2015-08-09

**Authors:** Zareh A. Grigoryan, Armen T. Karapetian

**Affiliations:** ^1^Goris State University, Avangard 4, 3204 Goris, Armenia; ^2^Yerevan State University of Architecture and Construction, Teryan 105, 0009 Yerevan, Armenia

## Abstract

The mutual influence of the slow rearrangements of secondary structure and fast collapse of the long single-stranded RNA (ssRNA) in approximation of coarse-grained model is studied with analytic calculations. It is assumed that the characteristic time of the secondary structure rearrangement is much longer than that for the formation of the tertiary structure. A nonequilibrium phase transition of the 2nd order has been observed.

## 1. Introduction

Single-stranded RNA (ssRNA) plays a central role in molecular biology. In addition to transmitting genetic information from DNA to proteins, RNA molecules participate actively in a variety of cellular processes. Examples are translation (rRNA, tRNA, and tmRNA), editing of mRNA, intracellular protein targeting, nuclear splicing of pre-mRNA, and X-chromosome inactivation. The RNA molecules involved in these processes do not code for proteins but act themselves as functional products. In addition, some RNA molecules prepared in vitro can bind to specific molecules such as ATP. In all these cases, the information encoded in the sequence of nucleotide bases of each RNA molecule determines its functional tertiary structure.

The forces which stabilize the secondary structure of RNA are stronger than interactions responsible for the tertiary structure and hence these two structures are characterized by two different energy scales. According to one of the currently accepted concepts of RNA folding the secondary structure elements, such as helices and loops hairpins, are formed first and then stack together to form a three-dimensional tertiary structure [1]. This is so-called hierarchical folding mechanism.

However, some experiments show that the folding rate of large RNA is lower than that predicted by the hierarchical mechanism [2]. This might mean that two successive folding steps are not fully independent. The landscape of the energy function of large RNA is extremely rugged and contains multiple deep minima which act as kinetic traps in the folding pathways [2]. The molecule can remain trapped in the states distinct from the native structure for time periods even longer than the average lifetime of RNA in a living cell [3, 4]. It should be noted that excellent models for describing the RNA secondary structure formation have been developed as well [5–10]. What remains relatively poorly understood is the full path of formation of the tertiary structure and, in particular, the mutual interplay between the secondary and tertiary structures [11–15].

The secondary structure of RNA is determined by the base pairing pattern. It has been shown that the characteristic pattern of the secondary structure of RNA is a tree-like structure, formed by relatively short double-stranded helices. The hierarchical folding scenario has been studied, for example, in [14], where the folding of RNA with fixed secondary structure is described by the model of tree-like polymer with quenched random branching. In [15] the concept of annealed randomly branched polymer has been applied to study the equilibrium characteristics of RNA. The completely annealed branching patterns describe the ensemble of secondary structures, wherein the tertiary structure is being formed as a result of substantial rearrangements of secondary structure elements. This scenario is typical for large RNAs, to which the hierarchical folding mechanism is most probably not applicable. While the model of a polymer with randomly annealed branching can be applied successfully for studying the equilibrium features of RNA folding, it cannot describe the folding kinetics efficiently. At the same time, the kinetic effects in the folding process are viewed to be of great importance due to the existence of long-living intermediates, as mentioned above.

In the present study we focus on the thermodynamic behaviour of the RNA molecule in the steady nonequilibrium state that occurs in case of well-defined separation between the relaxation timescales of secondary and tertiary structures. We introduce a reasonable coarse-grained model of RNA and study its behavior through analytical equations. The obtained results provide evidence for the existence of a nonequilibrium phase transition of the second order between the glassy phase and the ensemble of freely fluctuating spatial structures.

## 2. The Model

**Figure 1:**
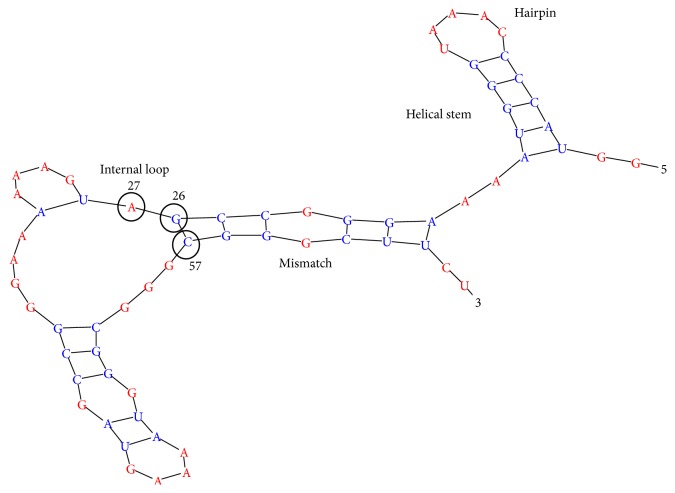
A fragment of the RNA chain with typical elements of secondary structure.

Let us consider the following mental experiment. The ssRNA molecule is dissolved initially in the solvent at temperature *T*′ which satisfies inequalities *θ* < *T*′ < *T*_*m*_, where *T*_*m*_ is the melting temperature and *θ* is the Flory temperature. Under these conditions the RNA molecule is a random coil with a well-defined secondary structure. Next, transfer a very small amount of our RNA containing solution into the same kind of solvent but with the temperature *T*, such that *T* < *θ*. In the beginning, the secondary and spatial structures in this state still correspond to the temperature *T*′ but they start to relax to the new temperature *T*. In the end of the process, the RNA will arrive at a compact globular state with some secondary structure pattern. The tertiary structure of RNA is stabilized by interactions between different elements of secondary structure: helical stems, hairpins, internal loops, mismatches, and so forth (Figure 1).

**Figure 2:**
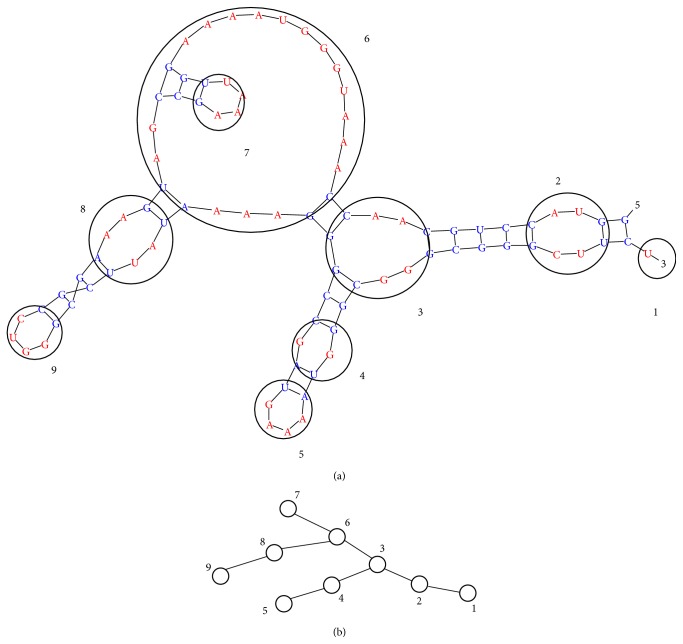
A fragment of the RNA secondary structure (a) and corresponding branched structure (b).

Interactions between helical stems can be considered as homogeneous since the nitrogen bases are located inside the double helix. At the same time, interactions between single-stranded regions are heterogeneous because of the interactions between nitrogen bases of different types. To describe the conformation of RNA in a coarse-grained approximation, we consider each nonpaired region of RNA as an effective monomer. The monomers are numbered by *i* = 1,…, *N*. The center of mass of monomer *i* is placed at the point with coordinates ${\vec{x}}_{i}$. Secondary structure is described in terms of the randomly branched polymer (Figure 2) as the matrix $\hat{B}=\left\|B_{ij}\right\|$, where *B*_*ij*_ = 1 if the *i*th and *j*th monomers are linked by helical stems and *B*_*ij*_ = 0, otherwise [14, 16]. The helices between the monomers are modelled as springs.

**Figure 3:**
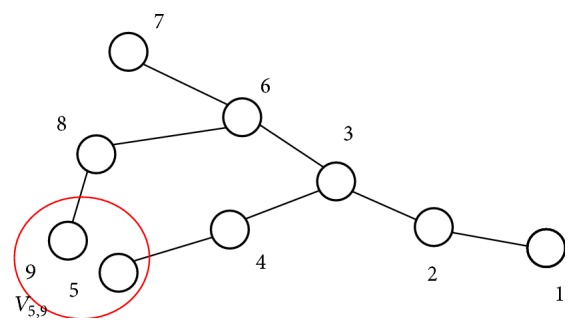
A coarse-grained model with monomers 5 and 9 interacting with the second virial coefficient *V*_5,9_.

Then we introduce the following Hamiltonian of the model:

(1) $$\begin{array}{c} H=H_{II}+\sum_{i<j}v_{ij}\delta \left(\;{\vec{x}}_{i}-{\vec{x}}_{j}\;\right)+V_{\text{conf}}, \end{array}$$

where the term $H_{II}\left(\left\{{\vec{x}}_{i}\right\},\hat{B}\right)=\left(dT/2\ell^{2}\right)\sum_{i<j}B_{ij}{\left({\vec{x}}_{i}-{\vec{x}}_{j}\right)}^{2}$ mimics the helical spring elasticity between the *i*th and *j*th effective monomers and *ℓ* is the equilibrium distance between neighbouring monomers which here coincides with the mean length of the helical stem. Thus, the helical spring elasticity constant is assumed to be equal to *dTℓ* ^2^. Here *v*_*ij*_ is the second virial coefficient of interaction between the *i*th and *j*th effective monomers, which refer to the tertiary contacts between nonpaired regions (loops). $V_{conf}\left(\left\{{\vec{x}}_{i}\right\}\right)$ is the confinement potential describing the homogeneous attraction between helical stems. The interactions between nonpaired regions (loops) *i* and *j* are governed by their size and nucleotide sequences (Figure 3).

Since many nucleotides contribute to the interaction of these effective monomers *i* and *j*, it is reasonable to consider coefficients *v*_*ij*_ as statistically independent random Gaussian variables with distribution

(2) $$\begin{array}{c} p\left(\;v_{ij}\;\right)\propto \exp\left(\;-\frac{v_{ij}^{2}}{2\Lambda^{2}}\;\right), \end{array}$$

where Λ is the variance of virial coefficients *v*_*ij*_. Collapse of the ssRNA molecule is driven mostly by electrostatic interactions and has been investigated experimentally [17, 18] and theoretically [10]. Unlike [10], here we do not take into account the counterions explicitly. Their impact is present implicitly in the confinement potential $V_{conf}\left(\left\{{\vec{x}}_{i}\right\}\right)=T\left(v_{0}\sum_{i<j}\delta \left({\vec{x}}_{i}-{\vec{x}}_{j}\right)+g_{0}\sum_{i<j<k}\delta \left({\vec{x}}_{i}-{\vec{x}}_{j}\right)\delta \left({\vec{x}}_{k}-{\vec{x}}_{j}\right)\right)$, which ensures existence of globular state of the RNA molecule. Here *v*_0_ < 0 and *g*_0_ > 0 are the second and third virial coefficients of interactions between helical stems, correspondingly.

Two types of conformational rearrangements in RNA are possible: rearrangement of the secondary structure with characteristic time scale *τ*_2_ and tertiary structure fluctuations with characteristic time scale *τ*_3_. The difference between timescales *τ*_2_ and *τ*_3_ is well pronounced. Thus as shown in [19] the collapse of 400-nucleotide-long RNA takes about 3-4 ms while the two-order shorter 21-nucleotide sequence of RNA folds into a hairpin in about 10 ms [20]. Therefore, in further calculations it will be assumed that *τ*_3_ ≪ *τ*_2_. The reason for this is not only the higher stability of base pairs as compared with the tertiary contacts. The kinetic factors play important role as well. Formation of the base pairs requires twisting of two single-stranded subchains into a double helix, which is kinetically hampered to unwind.

On the timescale *τ*, such that *τ*_3_ ≪ *τ* ≪ *τ*_2_, secondary structure and spatial arrangement of the effective monomers (nonpaired regions) are not in thermal equilibrium, and this stationary nonequilibrium steady state can be described in terms of the effective partition function [21]

(3) $$\begin{array}{c} Z={\langle \;{\langle \;{\left(\;Z_{\hat{B},\hat{v}}\;\right)}^{n}\;\rangle }_{\hat{B}}\;\rangle }_{\hat{v}}, \end{array}$$

where $Z_{\hat{B},\hat{v}}$ is the partition function of a branched molecule with the given branching pattern $\hat{B}$ and interaction matrix $\hat{v}=\left\|v_{ij}\right\|$. ${\langle \cdots \rangle }_{\hat{B}}$ means the average over all possible branching patterns, ${\langle \cdots \rangle }_{\hat{v}}$ is the average over intermonomer interactions, and *n* = *T*/*T*′. *T* and *T*′ are the effective temperatures of coarse-grained spatial and secondary structures correspondingly.

## 3. Thermodynamic Parameters

The effective partition function (3) is calculated by using the replica technique developed for systems with quenched disorder (see, e.g., [21, 22]). In our case the limit *n* → 0 corresponds to the quenched disorder, *n* = 1 describes the completely annealed disorder, and 0 < *n* < 1 for the partially annealed disorder.

Following the method described in [14] we rewrite the partition function (3) in the form

(4) $$\begin{array}{c} Z=\int D\rho e^{-\beta F\left\{\rho \right\}}, \end{array}$$

where $\beta =1/T,\;\;\rho \left(\vec{X}\right)=\sum_{i=1}^{N}\prod_{a=1}^{n}\delta \left({\vec{x}}_{i}^{a}-{\vec{x}}^{a}\right),\;\;\vec{X}=\left({\vec{x}}^{1},\ldots ,{\vec{x}}^{n}\right)$, and *F*{*ρ*} = *E*{*ρ*} − *TS*{*ρ*} is the *n*-replica free energy with

(5) Eρ=∑aVconfca−βΛ24∑a≠b∫dx dyqab2x→,y→Sρ≅−l42∫dXΔρX→2ρX→.

Here $c_{a}\left(\vec{x}\right)=\int dX\rho \left(\vec{X}\right)\delta \left({\vec{x}}^{a}-\vec{x}\right)$ is the one-replica density of monomers, $q_{ab}\left(\vec{x},\vec{y}\right)=\int dX\rho \left(\vec{X}\right)\delta \left({\vec{x}}^{a}-\vec{x}\right)\delta \left({\vec{x}}^{b}-\vec{y}\right)$ is the two-replica overlapping parameter, and Δ is the Laplace operator in the nd-dimensional space. Further in all equations we set *k*_*B*_ = 1. The energy term in (5) is obtained by averaging the *n*th power of the partition function over variables *v*_*ij*_, and the entropy term includes averaging over all possible branching patterns corresponding to the rooted tree with coordination number equal to three. For function $\rho \left(\vec{X}\right)$ the form $\rho \left(\vec{X}\right)=\rho_{0}exp\left(-\left(1/2\right)\sum k_{ab}{\vec{x}}^{a}{\vec{x}}^{b}\right)$ is used. The confinement potential is written as a virial expansion *V*_conf_(*c*) = *NT*((1/2)*v*_0_ *c* + (1/3!)*g*_0_ *c* ^2^). In compactly packed chain the density of monomers *c*_0_ ≈ *ℓ* ^−*d*^ and *V*_conf_(*c*_0_) ≈ *NT*(*v*_0_/4*ℓ* ^*d*^). Like in [22] we parameterize the offdiagonal entries of the matrix *k* by function *k*(*u*), where *u* ∈ [*n*, 1], and the diagonal entries as *k*_*aa*_ = *k*. The inverse matrix $\hat{m}={\hat{k}}^{-1}$ is parameterized by *m*(*u*), *u* ∈ [*n*, 1], and $m_{aa}=\tilde{m}=\ell^{2}N^{2/d}$. By introducing notations [*k*](*u*) = *uk*(*u*) − ∫_*n*_^*u*^ *dvk*(*v*) and *k* = ∫_*n*_^1^(*du*/*u* ^2^)[*k*](*u*), the free energy functional takes a form

(6) Fρ  =nVconfc0+βΛ44nN2πd2π−2d∫n1dum~2−mu2−d/2−Tl42dNnk2+∫n1duu2ku2−k~2.

## 4. Results and Discussion

Variation of the free energy (6) over $\tilde{k}$ and [*k*](*u*) as independent “variables” gives that the free energy per monomer has two branches:

(7) $$\begin{array}{c} f=\frac{F\left\{\rho_{0}\right\}}{Nn}=T\frac{v_{0}}{4l^{d}}+\left\{\begin{array}{c} f_{<},n\leq u_{0}\\ f_{>},n>u_{0} \end{array}\right\}, \end{array}$$

where the disordered free energy is

(8) $$\begin{array}{c} f_{<}=f_{<}^{0}-\frac{n^{\zeta }}{\zeta }\left(\;a_{1}-Tl^{4}dz_{0}^{2}\;\right), \end{array}$$

where *n* ≤ *u*_0_, *ζ* = (3*d* + 4)/(4 − *d*), and *a*_1_(*d*, *β*, Λ, *ℓ*) ∝ *β* ^−1^(*ββℓ* ^−*d*^)^1/*γ*(*d*)^. *f*_<_^0^ = *f*_<_^0^(*T*, *d*, Λ, *ℓ*) is the free energy of ssRNA with frozen secondary structure at *n* ≤ *u*_0_. At the same time, for *n* ≥ *u*_0_ the disordered free energy can be written as

(9) $$\begin{array}{c} f_{>}=f_{>}^{0}-n\left(\;a_{1}u_{0}^{\zeta -1}+Tl^{4}dz_{0}^{2}u_{0}^{\zeta +1}\frac{1}{n^{2}}\;\right), \end{array}$$

where *f*_>_^0^ = *f*_>_^0^(*T*, *d*, Λ, *ℓ*). Here the following notations are introduced: *z*_0_^2^(*d*, *β*, Λ, *ℓ*) ∝ (*β*Λ*ℓ* ^−*d*^)^1/*γ*(*d*)^, *γ*(*d*) = (4 − *d*)/8, *δ*(*d*) = (*d* + 4)/(4 − *d*), and *u*_0_(*d*) = (3/2)((3(*d*/2 + 1)+(*d* + 1)(*d*/2 − 1))/(*d* + 2)^2^) ≈ 0.6 if *d* = 3.

Thus, at *n* < *u*_0_ a glassy phase with replica-symmetry breaking is observed while at values *n* > *u*_0_ a replica symmetric phase is obtained.

The second derivatives of the free energy branches (8) and (9) with respect to variable *n* at the point of transition *n* = *u*_0_ satisfy equations

(10) ∂2f<∂n2n=u0=−ς−1u0ς−2a1−Tl4dz02∂2f>∂n2n=u0=−2u0ς−2Tl4dz02

while the first derivatives are as follows: (∂*f*_<_/∂*n*)|_*n*=*u*_0__ = (∂*f*_>_/∂*n*)|_*n*=*u*_0__ = −*u*_0_^*ς*−1^(*a*_1_ − *Tℓ* ^4^ *dz*_0_^2^). Thus, in the nonequilibrium state with 0 < *n* < 1 the model exhibits nonequilibrium phase transition of the second order. The temperature of transition is

(11) $$\begin{array}{c} T_{c}^{\prime }=\frac{T}{u_{0}}. \end{array}$$

In the framework of the proposed model the value of the parameter 1 − *n* serves a measure of the distance from the equilibrium state, corresponding to the *n* = 1. If RNA molecule is far enough from the equilibrium (*n* < *u*_0_), then the glassy phase is realized which is dominated by a few long-lived intermediates, which were observed experimentally in [23].

## 5. Conclusion

The interplay between the secondary structure formation and fast collapse of the single-stranded RNA is addressed in terms of the model with interaction between heterogeneous nonpaired and homogeneous double-stranded regions. Thus, the nucleotide sequence heterogeneity is approximately described in terms of statistical-mechanical model with disorder [22]. The memory effects in RNA compact structure formation are governed by slow rearrangements of the secondary structure with subsequent fast relaxation of the spatial degrees of freedom. Under these conditions, the mutual equilibration of fast and slow variables is hindered. Monomers rapidly attain their equilibrium at the temperature *T* and thus, because of the wide timescale gap, their equilibrium free energy acts as a driving force pushing the slow dynamics of the elements of secondary structure to reach a nonequilibrium stationary state at long times. This scheme is known generally as the adiabatic elimination of fast variables [24–26]. The observed experimentally [23] long-lived intermediates are obtained if RNA molecule is far enough away from the state of thermodynamic equilibrium.
